# Fuzzy Modeling Development for Lettuce Plants Irrigated with Magnetically Treated Water

**DOI:** 10.3390/plants12223811

**Published:** 2023-11-09

**Authors:** Fernando Ferrari Putti, Camila Pires Cremasco, Alfredo Bonini Neto, Ana Carolina Kummer Barbosa, Josué Ferreira da Silva Júnior, André Rodrigues dos Reis, Bruno César Góes, Bruna Arruda, Luís Roberto Almeida Gabriel Filho

**Affiliations:** 1 School of Science and Engineering, São Paulo State University (UNESP), Tupã 01049-010, SP, Brazil; camila.cremasco@unesp.br (C.P.C.); alfredo.bonini@unesp.br (A.B.N.); andre.reis@unesp.br (A.R.d.R.); bruna.arruda@unesp.br (B.A.); gabriel.filho@unesp.br (L.R.A.G.F.); 2 Department of Civil Engineering, Ponta Grossa State University (UEPG), Ponta Grossa 84010-330, PR, Brazil; ackummer@hotmail.com; 3 Department of Agronomy, Federal University of Triângulo Mineiro (UFMT), Iturama 38280-000, MG, Brazil; josue.junior@uftm.edu.br; 4 Department for Business, Adamantina College of Technology (FATEC), Adamantina 17800-000, SP, Brazil; bruno.goes5@fatec.sp.gov.br

**Keywords:** *Lactuca sativa*, growth, uncertain, precision, curves

## Abstract

Due to the worldwide water supply crisis, sustainable strategies are required for a better use of this resource. The use of magnetic water has been shown to have potential for improving irrigation efficacy. However, a lack of modelling methods that correspond to the experimental results and minimize error is observed. This study aimed to estimate the replacement rates of magnetic water provided by irrigation for lettuce production using a mathematical model based on fuzzy logic and to compare multiple polynomial regression analysis and the fuzzy model. A greenhouse study was conducted with lettuce using two types of water, magnetic water (MW) and conventional water (CW), and five irrigation levels (25, 50, 75, 100 and 125%) of crop evapotranspiration. Plant samples for biometric lettuce were taken at 14, 21, 28 and 35 days after transplanting. The data were analyzed via multiple polynomial regression and fuzzy mathematical modeling, followed by an inference of the models and a comparison between the methods. The highest biometric values for lettuce were observed when irrigated with MW during the different phenological stage evaluated. The fuzzy model provided a more exact adjustment when compared to the multiple polynomial regressions.

## 1. Introduction

Lettuce (*Lactuca sativa*) is one of the most consumed vegetables worldwide, with highlighted economic and social impact due to production and commercialization by small farmers. Recently, the consumption of lettuce has increased due to extensive use in fast-food restaurants as an ingredient [1] and due to its nutritional properties as a source of vitamins and minerals [2,3], with a low calorie content.

In terms of production, lettuce needs a large water supply for the whole cycle to guarantee adequate development, as lettuce is highly sensitive to water deficiency. Thus, in regions with marked water scarcity, techniques such as reuse of wastewater have been used and have provided satisfactory results [4]. Water deficit stress reduces the root and shoot biomass, causing a reduction in the photosynthesis process, hence compromising sugar metabolism, which is the main energy source for plant development and ATP formation [5,6].

On the other hand, in regions with irrigation availability, techniques for optimizing irrigation have been researched to reduce wastage [7,8], to avoid the misuse of water resources [9], and to adjust the amount of electricity for the pressurization of the system. Studies using magnetically treated water in irrigation systems have demonstrated incremental improvement in the soil moisture, a reduction in the volume of water applied and incremental improvement in plants’ nutrient uptake, resulting in yield gains for many crops [10,11]. Additionally, in high-tech areas, the implementation of precision irrigation generates high efficiency and extremely low waste [12]. In China, Zhang et al. [13] observed a possible reduction of about 20% in total water consumption irrigation with improvement in efficiency.

To this end, models have been used to predict the actual effects of irrigation systems on crops, supported by experimental assays to reduce error. Multiple polynomial regression analysis emphasizes the explanation of each variable to the model [14]. This is a widespread technique and frequently applied to agricultural sciences. However, the responses of equations derived from statistical analysis may generate built-in error [15,16]. Therefore, further studies with mathematical models are required to reduce error and enhance the accuracy of the models.

Among the mathematical models, the fuzzy model, in which the truth values of the variables can be any real number between 0 and 1, has been highlighted to predict inaccurate phenomena with greater accuracy, such as potato yield prediction [17], the vitality of orchids [18], the automatization of water flow control in pipes [19], precision sensors [20] and control of agricultural spraying [21,22]. Additionally, fuzzy models present a variety of applications in expert systems that search for algorithm knowledge conversion, such as the selection of wheat genotypes [23] and management of cotton crops [24].

Fuzzy modeling applied to the evaluation of irrigation system performance has been verified and better responses reported compared to standard models [25,26,27,28], such as multiple polynomial regression analysis. Based on this, Giusti and Marsili-Libelli [29] have developed a support system based on fuzzy logic for irrigation and water conservation in agriculture.

The present study aims: (i) to estimate, using a mathematical model based on fuzzy logic, values between the maximum and minimum for the rates of magnetically treated water replacement provided via irrigation for lettuce production and (ii) to compare the results of multiple polynomial regression analysis and the fuzzy model results.

## 2. Materials and Methods

### 2.1. Experiment Description

The experiment was conducted at the Department of Agricultural Engineering at Sao Paulo State University (UNESP), located in Botucatu, São Paulo, from January to April 2012.

The experiment was set up in the ground of a greenhouse, with soil classified, according to Santos et al. [30], as Nitossolo Red Dystrophic latosolic, with moderate clay structure and physical and chemical characteristics (0 to 20 cm depth) as presented: pH_CaCl2_ 5.9; organic matter 24 g dm^−3^; P (Resin) 191 mg dm^−3^; H + Al 17 mmol dm^−3^; K 4.8 mmol dm^−3^; Ca 68 mmol dm^−3^; Mg 25 mmol dm^−3^; sum of base (SB) 97 mmol dm^−3^; cation exchange capacity (CEC) 114 mmol dm^−3^; base saturation index (V%) 85%; B 0.51 mg dm^−3^; Cu 4.8 mg dm^−3^; Fe 14 mg dm^−3^; Zn 8 mg dm^−3^; sand 37%; silt 51%; clay 12%; and soil density 1.21 g cm^−1^.

Two crop cycles (35 days long), namely the 1st and 2nd cycle, of lettuce (variety Veronica) were performed. The lettuce cultivation occurred in ten flowerbeds, each with a 1.2 m length and a 1.0 m width, totaling 3.6 m^2^ in area. The spacing used was 0.25 between lines and 0.25 between plants, totaling 40 plants.

Moreover, a randomized block design was adopted, in a 5 × 2 factorial: five irrigation levels (25, 50, 75, 100 and 125% crop evapotranspiration—ETc), during the whole of both cycles; and two sources of water: either magnetically treated water (MW) or conventional water (CW) with no treatment.

**Figure 1:**
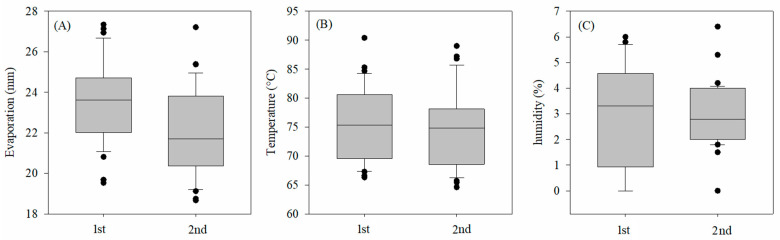
Monitoring of (**A**) evaporation (mm); (**B**) temperature (°C); and (**C**) humidity (%) for the duration of the 1st cycle and 2nd cycle of lettuce plants, in a greenhouse.

The estimation of evaporation of the culture was obtained using the class-A tank coefficient of the culture (Kc) and the tank coefficient (Kp), which disregarded wind speed. To determine the lettuce ETc, a class-A tank was installed inside of the greenhouse, and an automatic weather station collected and stored data regarding daily temperature and humidity (Figure 1).

To obtain the MW, a magnetizer (Sylocymol Rural), with the capacity to magnetize 5 m^3^ of water every 30 min, was inserted into the MW reservoir (500 L capacity).

**Figure 2:**
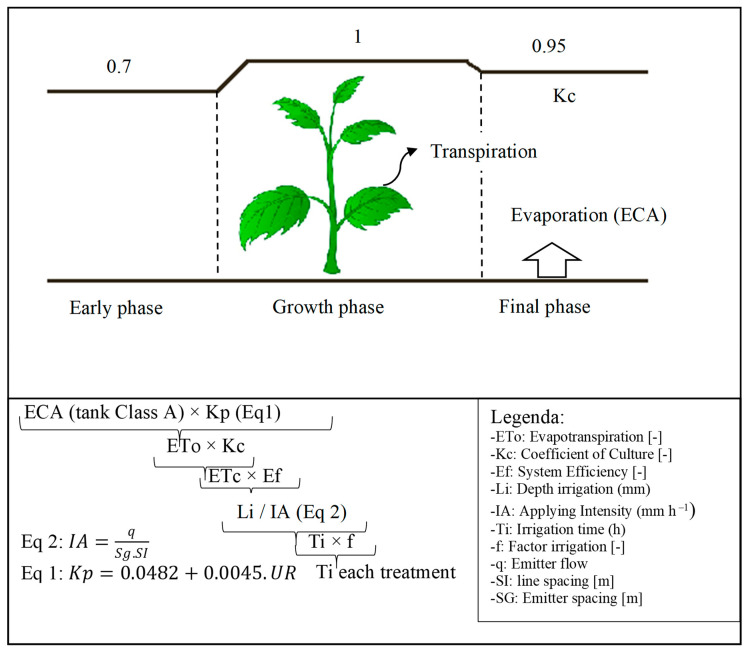
Method of determining evapotranspiration and method adopted for calculating water depth and irrigation time.

Two independent dripping irrigation systems were implemented, one for each type of water (MW and CW). The water amount varied according to the lettuce cycle (Figure 2).

Four samplings were performed throughout each lettuce cycle, at 14, 21, 28 and 35 days after transplanting (DAT), respectively, following biometric parameters, with 4 replicates, disregarding the plants located on the borders of the plots. In these samplings, the roots and shoots of lettuce plants were separated using a stylus. The leaf number (LN) was counted. Fresh shoot and root biomass, FSB and FRB, respectively, were obtained using a digital balance. Dry shoot and root biomass, DSB and DRB, respectively, were obtained after keeping the samples in an oven (65 °C) until constant weight was reached. Afterwards, each sample was weighed.

### 2.2. Preliminary Analysis of Data

A test of normality was performed on each data set using the Anderson–Darling test. For the data set with homoscedasticity, the constant variance of the data error was analyzed using the equation of variance test and Bartlett’s test [31]. For the data sets without normal distribution and/or with differences in variance, a Box–Cox test was used [32], and the data were normalized with Equation (1):

(1) $$y_{i}=\frac{x_{i}^{\lambda }-1}{\lambda },\;\lambda \neq 0$$

wherein $x_{1},\;\ldots ,\;x_{n}$ are the original data; $y_{i}$ is the approximate data to the normal distribution and λ is the parameter of data transformation.

### 2.3. Multiple Polynomial Regression Analysis

A model was predicted using known independent variable data $\left(x_{1},\;x_{2},\;x_{3},\;\ldots ,\;x_{k}\right)$ to estimate the value of the dependent variable (y) [33,34], wherein the general model can be given by Equation (2):

(2) $$y=\beta_{0}+ax_{1}+a_{1}x_{2}+a_{2}x_{2}^{2}+a_{3}x_{2}^{3}$$

wherein *β*_0_ is the linear coefficient; $x_{1}$ is the type of water treatment ($x_{1}=0$, for CW and $x_{1}=1$ for MW), $x_{2}$ is the percentage of ETc, adopted as irrigation level ${(x}_{2}\in [25;125\%])$.

After calculating the adjustment equation for the dependent variables (y) (biometric responses) in the function of the independent variables ($x_{1}$ and $x_{2}$), the coefficient of determination $\left(R^{2}\right)$ was calculated to determine the adjustment intensity.

The F test (F statistic) was performed to verify if the equation presents a certain degree of confidence and if the relationship established between the dependent variable (x) and the independent variables (y) is relevant. The *p* value for each equation was obtained to verify the significance of the model [14,35].

### 2.4. Elaboration of a Model Based on the Fuzzy Logic

The proposed fuzzy mathematical modeling used intervals at the factor levels, namely $\left[25k\%,25\left(k+1\right)\%\right],1\leq k\leq 4$. At level 25, k% 1≤c≤4 was evaluated throughout the samplings (14, 21, 28 and 35 DAT), using analogous modeling for each cycle, for leaf number (LN), fresh shoot biomass (FSB), fresh root biomass (FRB), dry shoot biomass (DSB), and dry root biomass (DRB), as in the agronomic characteristics model: $f:{X_{1}\times X_{2}\subset R}^{2}\to R^{5}$, where $X_{1}$ is water type (MW or CW) and $X_{2}$ is irrigation level (25, 50, 75, 100 and 125% of ETc), with $y=f(\bar{x}$), wherein R is the set of real numbers; x=($x_{1},x_{2})$; $x_{1}=irrigationlevel$, with $x_{1}\in X_{1}=\{25,125\}$; $x_{2}=$ days after transplanting, with $x_{2}\in X_{2}=[35]$ and $y=(y_{1},\ldots .,y_{5})$ defined by the mean values of the biometric characteristics, namely $y_{1}=\bar{LN}$, $y_{2}=\bar{FSB}$, $y_{3}=\bar{FRB}$, $y_{4}=\bar{DSB}$ and $y_{5}=\bar{DRB}$.

This system was based on fuzzy rules and follows the function $F\left[25,125\right]\times \left[14,35\right]\to R^{5},F\left(x,y\right)=\left(f_{1}\left(x,y\right),f_{2}\left(x,y\right),f_{3}\left(x,y\right),f_{4}\left(x,y\right),f_{5}\left(x,y\right)\right)$, where the Cartesian product that represents the area of the evaluations during the cycle (14 to 35 DAT) and irrigation levels (25 to 125% of ETc), and the codomain $R^{5}$ represent the five response variables evaluated in the experiment.

The data sets were analyzed in two responses surfaces; one for each type of water (MW and CW) and another one for each lettuce cycle (1st and 2nd) for the graph function as follows:

- Group 1: $F_{1}^{0}:\left[14,35\right]\times \left[25;125\right]\to R,F_{1}^{0}\left(0,y\right)=f_{1}(0,y)$, wherein the codomain of $F_{1}^{0}$ is relative to the leaf number;$\;F_{1}^{1}:\left[14,\;35\right]\times \left[25;125\right]\to R,F_{1}^{1}\left(0,y\right)=f_{1}(1,y)$, wherein the codomain of $F_{1}^{1}$ is related to leaf number;
- Group 2: $F_{2}^{0}:\left[14,35\right]\times \left[25;125\right]\to R,F_{2}^{0}\left(0,y\right)=f_{2}(0,y)$, wherein the codomain of $F_{2}^{0}$ is related to the fresh shoot biomass; $F_{2}^{1}:\left[14,\;35\right]\times \left[25;125\right]\to R,F_{2}^{1}\left(0,y\right)=f_{2}(1,y)$, in which the codomain of $F_{2}^{1}$ is related to fresh shoot biomass;
- Group 3: $F_{3}^{0}:\left[14,35\right]\times \left[25;125\right]\to R,F_{3}^{0}\left(0,y\right)=f_{3}(0,y)$, wherein the codomain in $F_{3}^{0}$ is related to dry shoot biomass;${\;F}_{3}^{1}:\left[14,\;35\right]\times \left[25;125\right]\to R,F_{3}^{1}\left(0,y\right)=f_{3}(1,y)$, in which the codomain of $F_{3}^{1}$ is related to dry shoot biomass;
- Group 4: ${\;F}_{4}^{0}:\left[14,35\right]\times \left[25;125\right]\to R,F_{4}^{0}\left(0,y\right)=f_{4}(0,y)$, wherein the codomain in $F_{4}^{0\;}$ is relative to the fresh root biomass $F_{4}^{1}:\left[14,\;35\right]\times \left[25;125\right]\to R,F_{4}^{1}\left(0,y\right)=f_{4}(1,y)$, wherein the codomain of $F_{4}^{1}$ is related to fresh root biomass;
- Group 5: ${\;F}_{5}^{0}:\left[14,35\right]\times \left[25;125\right]\to R,F_{5}^{0}\left(0,y\right)=f_{5}(0,y)$, wherein the codomain of $F_{5}^{0}$ is related to fresh root biomass;$\;F_{5}^{1}:\left[14,\;35\right]\times \left[25;125\right]\to R,F_{5}^{1}\left(0,y\right)=f_{5}(1,y)$, wherein the codomain of $F_{5}^{1}$ is related to dry root biomass.

**Figure 3:**
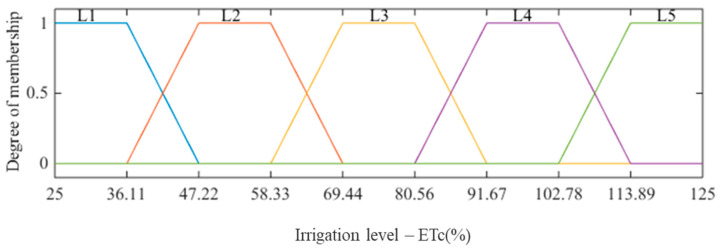
Degree of membership functions defined for five fuzzy sets (*L_i_*) for input variable irrigation level ({25, 125}% of Etc).

For the input variable “irrigation level”, five fuzzy sets $\left(L_{i}\right)$ were considered, denoted as $L_{i},i=1,2,3,4,5$, following the five dimensioned irrigation levels used according to ETc:$\;\left(25i\right)\%,i=1,2,3,4,5$ in the lettuce cultivation. Pertinence trapezoidal functions were adopted from the sets $L_{i}$, in accordance with Yet [36], due to the best model adjustments in the answer model for a set of data presenting a continuous variable. These functions were defined as if any irrigation rate had a pertinence grade equal to 1 set, so that each ratio of ETc (%) had a degree of pertinence equal to the corresponding fuzzy set $\left(u_{L_{i}}\left(25i\%\right)=1\right)$ and $u_{L_{i}}\left(x\right)=1,x_{i-1}\leq x\leq x_{i+1},$ with $x_{i+1}-x_{i-1}=k$, to a certain k, in which 9k=100%, according to the adopted relevance function (Figure 3).

Thus, Equation (3) was used to determine the delimiters’ relevance (k), aiming a symmetrical variation between the delimiters:

(3) $$k=\frac{x_{\max}-x_{\min}}{2n-1}\Rightarrow k=\frac{125\%-25\%}{9}=11.11\%$$

wherein $x_{\max}$ is the maximum rated point; $x_{\min}$ is the estimated minimum point; and n is the number of fuzzy sets.

From the five fuzzy sets relating to the irrigation level, a variation of 11.11% was obtained. The sizing of each delimiter was represented generically by 25+n.k,n=0,1,…,9, where for the lower and upper delimiter, the subtraction of k on the bounding $x_{1}$ of the first fuzzy set and the addition of k in the enclosing $x_{4}$ of the last fuzzy set were adopted. Therefore, nine delimiters needed to be calculated, in which the variation was 11.11%.

**Figure 4:**
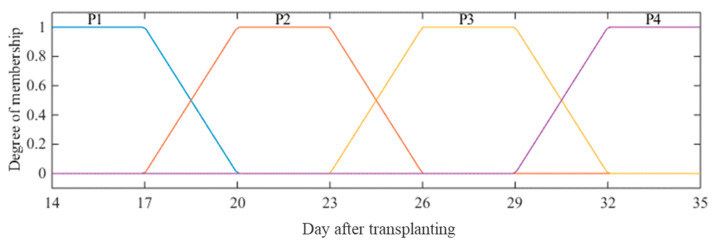
Degree of membership functions defined for four fuzzy sets (*P_i_*) for the input variable days after transplanting (DAT). P1: fuzzy set of DAT 14; P2: fuzzy set of DAT 21; P3: fuzzy set of DAT 28; P4: fuzzy set of DAT 35.

For the input variable “samplings; days after transplanting—DAT”, four fuzzy sets ${\;(P}_{i})$ were considered, denoted as $P_{i}$, i=1,2,3,4, referring to the four sampling times during the lettuce cycling: 14+7i, i=0,1,2,3. The pertinence functions of the trapezoidal type were implemented for the sets $P_{i}$. Thus, the functions determined for each sampling (DAT) had a degree of pertinence equal to 1 for their fuzzy sets (Figure 4).

Equation (4) was used for determining the delimiters from the pertinence (k) to obtain a symmetrical variation between the delimiters:

(4) $$k=\frac{x_{\max}-x_{\min}}{2n-1}\Rightarrow k=\frac{35-14}{7}=3$$

wherein $x_{\max}$ is the maximum rated point; $x_{\min}$ is the estimated minimum point; and n is the number of fuzzy sets.

**Figure 5:**
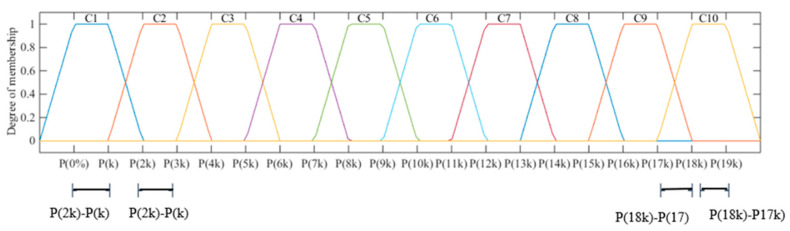
Degree of membership functions defined for ten fuzzy sets (*C_i_*) for the input variable: leaf number (LN); fresh shoot root biomass (FSB); fresh root biomass (FRB); dry shoot biomass (DSB) and dry root biomass (DRB). C1–C10: refers to the generic pertinence sets for the output variables that have been modeled using fuzzy logic.

Therefore, the use of five variables (LN, FSB, FRB, DSB and DRB), for both lettuce cycles (1st and 2nd) led to setting up ten fuzzy sets ${\;(C}_{i})$:${\;C}_{n}$, m=1,2,…,10, with trapezoidal pertinence functions. Nineteen delimiters were adopted as the percentage of the measured data sets of each output variable to enable the set of trapezoidal shape of the ten sets of relevance. This percentage, denoted as P(x%), depends on the constant k, since the required delimiters are in the form P(mk) 0≤m≤18 (Figure 5).

The constant *k* was calculated by Equation (5):

(5) $$k=\frac{x_{\max}-x_{\min}}{2m-1}\Rightarrow k=\frac{100\%-0\%}{19}=5.26\%$$

wherein $x_{\max}$ is the maximum peak observed for the output variables; $x_{\min}$ is the minimum point observed for the output variable; and m is the number of fuzzy sets.

Therefore, for definition of relevant and output variable function, 19 percentiles with a range of 5.26% were used, creating pertinence function for the output variables in the proposed methodology, where for the $x_{1}$ in the fuzzy set, C1 was subtracted from the *k* value and the *x*_4_ delimiter fuzzy *C*10 set was added to the *k* value.

The development of the fuzzy system assumed the fuzzy base rule:

- If “premise (antecedent)” then “conclusion (consequent)”;

From the input variables, twenty pairs of rules (5 irrigation level × 4 samplings) were created and associated with five output variables (LN, FSB, FRB, DSB and DRB) (Table 1). The base rule created for the proposed fuzzy model was determined according to the methodologies proposed by Putti, Cremasco et al. and Gabriel Filho et al. [18,37,38].

Associations of the output variable (S) fuzzy sets were used to calculate the output variable values with relevance degree 1. Due to the need to calculate 19 delimiters, the percentiles of level 0% (minimum) and 5.36.i, with i=1,2,…,18,19 of the output variables data (S) were determined, enabling the subsequent classification of the output variable from the points with pertinence degree 1, characterized by the base rule of fuzzy systems:

- If S≤P(5.26%) so VS is “C1”;
- If $P\left(10.52\%\right)\leq S\leq P(15.78\%)$ so VS is C2;
- If $P\left(21.04\%\right)\leq S\leq P(26.3\%)$ so VS is C3;
- If $P\left(31.56\%\right)\leq S\leq P(36.82\%)$ so VS is C4;
- If $P\left(42.08\%\right)\leq S\leq P(47.34\%)$ so VS is C5;
- If $P\left(52.6\%\right)\leq S\leq P(57.86\%)$ so VS is C6;
- If $P\left(63.12\%\right)\leq S\leq P(68.38\%)$ so VS is C7;
- If $P\left(73.64\%\right)\leq S\leq P(78.9\%)$ so VS is C8;
- If $P\left(84.16\%\right)\leq S\leq P(89.42\%)$ so VS is C9;
- If $VS\geq P\left(94.68\%\right)$ so VS is C10,

Wherein P (x %) is the percentage level of all calculated output variables values and S is the output variable, with S∈{LN, FSB, FRB, DSB and DRB}.

### 2.5. Inference of the Fuzzy Method

Once the propositions of the antecedent and consequent of the proposed model are fuzzy, an inference method can be used as proposed by Mamdani and Assilian [39], and according to Ross [40], this is the most common method cited in literature. For this, the air center or centroid method [40,41,42,43,44,45,46,47,48,49,50] was used with Equation (6):

(6) $$y=\frac{\sum_{x}\mu_{a}\left(x\right)x}{\sum_{x}\mu_{a}\left(x\right)}$$

wherein $x_{\max}$ is the maximum peak observed for the output variables; $x_{\min}$ is the minimum point observed for the output variable; and n is the number of fuzzy sets.

### 2.6. Analysis of the Fuzzy Model Association Intensity

To determine the intensity degree of the models obtained in the lettuce experiment, three tests were used:

#### 2.6.1. Medium Square Error

(7) $$MSE=\sum_{i=1}^{n}\frac{{\left(y_{observed}-y_{fuzzy}\right)}^{2}}{n}$$

#### 2.6.2. Pearson’s Correlation (*r*)

(8) r=∑i=1n(yfuzzy−y¯fuzzy)(yobserved−y¯observed)[∑i=1n(yfuzzy−y¯fuzzy)2(yfuzzy−y¯fuzzy)2]12

#### 2.6.3. The Willmott et al. [51] Index

(9) $$d=1-\left[\frac{\sum_{i=1}^{n}{\left|y_{fuzzy}-y_{observed}\right|}^{2}}{\sum_{i=1}^{n}{\left(\left|y_{fuzzy}-\bar{y}\right|+\left|y_{observed}-\bar{y}\right|\right)}^{2}}\right]$$

wherein $\bar{y}$ is the average of values and n is the number of fuzzy sets.

These methodologies allowed us to compare the adjustment curves established in this study, determined via multiple polynomial regression analysis and the proposed model based on fuzzy logic. To determine the maximum score, a simulation within the established fuzzy system was carried out, with variations steps of 0.1 for irrigation levels and sampling (DAT), to confirm the condition that resulted in the best performance.

### 2.7. Software Used

Matlab software, version 8.4 (2014), was used for the development of the systems based on fuzzy rules and for the simulation of variable responses. The simulated data were plotted in a spreadsheet using Excel software 2016 (Microsoft Office). Minitab statistical software, version 17.0 (2014), was used for calculating the multiple regression model.

## 3. Results

### 3.1. Multiple Polynomial Regression Adjustment

The setting equations using multiple polynomial regression adjustment, in general, occurred as 3rd degree equations (Table 2).

### 3.2. Developed Model Based on the Fuzzy Logic

The pertinence functions associated the highest degree of pertinence for each evaluation of the lettuce crop, when submitted to irrigation levels (25, 50, 75, 100 and 125% of crop evapotranspiration—Etc), with two types of water, MW and CW, sampled four times (14, 21, 28 and 35 days after transplanting—DAT) were defined as similar for each cycle (1st and 2nd). Then, the fuzzy set of biometric variables was developed. The following statements represent the relationships found in the construction model with other analogous outputs.

- If (DAT is “P1”) and (irrigation level is “L1”), then (NL is “C2”, FSB is “C3”, DSB is “C3”, FRB is “C3” and DRB is “C2”);
- If (DAT is “P1”) and (irrigation level is “L2”), then (NL is “C2”, FSB is “C3”, DSB is “C3”, FRB is “C6” and DRB is “C2”);
- If (DAT is “P1”) and (irrigation level is “L3”), then (NL is “C3”, FSB is “C3”, DSB is “C3”, FRB is “C8” and DRB is “C3”).

The rules presented above are the commands for the fuzzy controller, which, with reference to the DAT and Irrigation Blade, will perform the calculation to predict the results of the biometric components.

**Figure 6:**
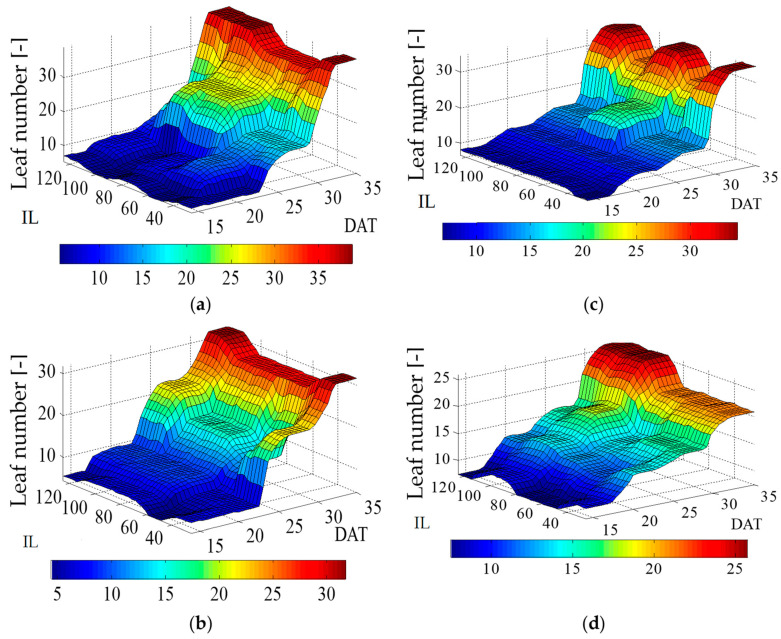
Lettuce leaf number in response to different irrigation levels (IL) (25, 50, 75, 100 and 125% of crop evapotranspiration—ETc), and two types of water magnetically treated water (MW) and conventional water (CW), sampled at 14, 21, 28 and 35 days after transplanting—DAT, for two cycles (1st and 2nd): (**a**) 1st cycle—MW; (**b**) 2nd cycle—MW (**c**) 1st cycle—CW (**d**) 2nd cycle—CW.

The construction methodology of the output fuzzy model function enables the vertex determination of the graphic (Figure 6).

The analysis of the leaf number shows that the lettuce irrigated with magnetically treated water (MW) showed higher plant development compared to the plants irrigated with conventional water CW (Figure 6). A larger number of accumulated leaves occurred during the 1st cycle, when the lettuce was irrigated with MW, and with irrigation rates at 75% and 100%, where the lettuce leaf number was lower, with similar pattern between th^e^ 1st and 2nd cycle (Figure 6a,b). On the other hand, when the lettuce was irrigated with CW, less development over the cycle was noted, with plants accumulating 32 leaves at the end of the 1st cycle (Figure 6c), and a weaker development over the 2nd cycle was observed, producing a maximum of 26 leaves when subjected to irrigation at 125% of Etc.

**Figure 7:**
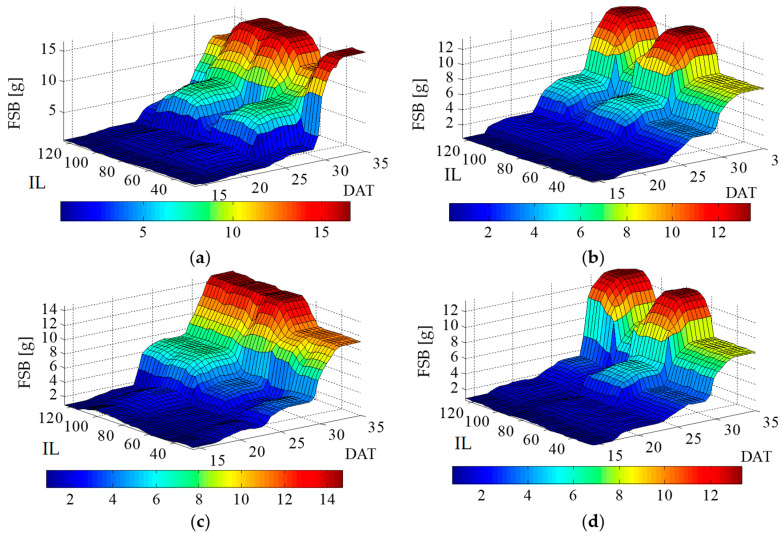
Lettuce fresh shoot biomass in response to different irrigation levels (IL) (25, 50, 75, 100 and 125% of crop evapotranspiration—ETc), and two types of water (magnetically treated water (MW) and conventional water (CW), sampled at 14, 21, 28 and 35 days after transplanting—DAT, for two cycles (1st and 2nd): (**a**) 1st cycle—MW; (**b**) 2nd cycle—MW; (**c**) 1st cycle—CW; (**d**) 2nd cycle—CW.

For fresh shoot biomass (FSB), in general, high values were observed in response to irrigation with MW (Figure 7). When the 1st cycle was analyzed, when lettuce was irrigated with MW, weaker development occurred, with a high FSB value occurring close to the replacement rate of 100% of ETc (Figure 7a). When lettuce plants were irrigated with CW, a lower FSB was observed in comparison to plants irrigated with MW. The maximum FSB, with CW, occurred near the irrigation rates of 125 and 75% of ETc (Figure 7b). During the 2nd cycle, the same pattern was observed. The highest FSB was observed when lettuce plants were irrigated with MW, at the irrigation rate of around 125% of ETc (Figure 7b). When the lettuce was irrigated with CW, an effect similar to that of the 1st cycle was observed, with greater accumulation of FSB at replacement rates of 75 and 125% of ETc (Figure 7d).

Fresh root biomass (FRB) showed higher values when lettuce was subjected to irrigation with MW, with similar trend over both cycles. Irrigation with MW presented the best FRB accumulation when plants were irrigated at rates of 75% and 100% of ETc during both cycles (Figure 7a,b).

**Figure 8:**
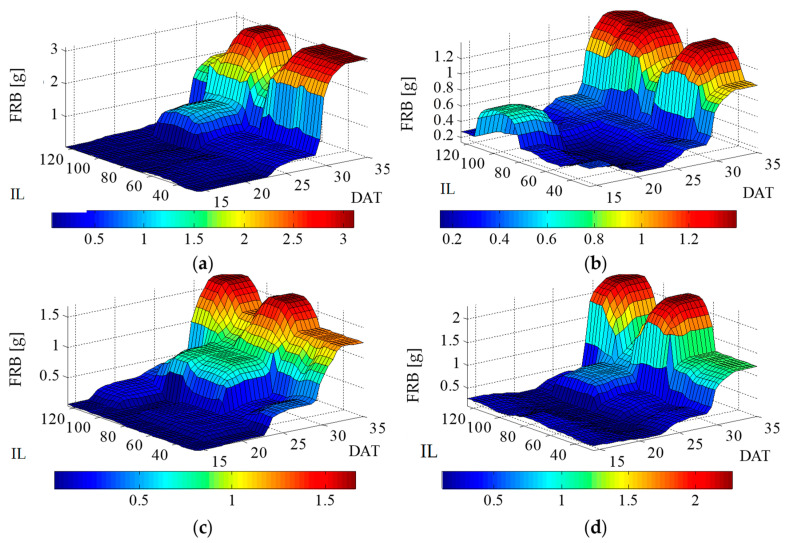
Lettuce fresh shoot biomass in response to different irrigation levels (25, 50, 75, 100 and 125% of crop evapotranspiration—ETc), and two types of water (magnetically treated water (MW) and conventional water (CW), sampled at 14, 21, 28 and 35 days after transplanting—DAT, for two cycles (1st and 2nd): (**a**) 1st cycle—MW; (**b**) 2nd cycle—MW (**c**) 1st cycle—CW (**d**) 2nd cycle—CW.

Like FSB, the accumulation of dry shoot biomass (DSB) followed a trend where the highest values were observed in plants irrigated with MW. In both cycles (1st and 2nd), large increments occurred with replacement irrigation rate close to 100% of ETc (Figure 8a,b). When lettuce was irrigated with CW, a similar effect in both cycles was observed, where the replacement rate of 50% of ETc differed from the other rates at the end of the cycles (Figure 8c,d).

Regarding irrigation with CW, the behavior was the same over both cycles, where regions close to the replacement rates of 75% and 100% of ETc obtained the highest accumulations of dry shoot biomass (Figure 8c,d). Dry root biomass (DRB) followed the same trend as FRB (Figure 7).

It is possible to infer that in th^e^ 1st cycle, when plants were submitted to irrigation with MW, higher accumulation occurred close to the replacement rates of 50 and 100% of ETc (Figure 8a). As for the 2nd cycle, the highest increase occurred when plants were irrigated at the replacement rate of 50%, 100% and 125% of ETc (Figure 8b).

Irrigation with CW had a similar effect over both cycles, in which we can highlight the higher development obtained when replacement rates were between 75% and 125% of ETc (Figure 8c,d).

### 3.3. Analysis of the Models Association Intensity

The developed fuzzy model and the multiple polynomial regression equations used in this study permitted us to verify the degree of association in the models with the collected data from the lettuce experiment, which were used as parameters for correlation analysis via mean square error (MSE), Pearson’s correlation (r), and the Willmott et al. [51] index (d) (Table 3).

The correlation results suggests that the fuzzy models were more accurate than multiple polynomial regression, as fuzzy models showed greater correlation for the data from the lettuce experiment and more accurate adjustments.

## 4. Discussion

The biometric parameters of lettuce observed in the experiment enables us to infer that the use of magnetically treated water promoted a better development of the lettuce. Once water is submitted to a magnetic field, changes to its properties are observed, such as increased in water absorption in surfaces [52], solubility of some minerals [53,54,55,56], surface tension [57], crystallization and precipitation of salts [58,59]. Additionally, Mostafazadeh et al. and Khoshnevisan et al. [10,17] observed incremental changes in soil moisture by using magnetized water compared to nonmagnetic water. All these changes together generate benefits to different crops.

The increase in production parameters found in lettuce irrigated with magnetically treated water corroborates several surveys that describe technology with a high potential of reducing the volume of water applied, such as in the cultivation of celery, green beans and peas [60]; chickpeas [61]; castor beans [62]; corn [63]; tobacco [64]; peas [65]; tomatoes [66,67]; wheat [68]; and pepper [69]. Putti et al. [70] also reported the beneficial effects of magnetically treated water on lettuce plants due to improvement in nutritional status and yield.

However, when lettuce was irrigated with magnetically treated water, among the parameters analyzed, the leaf number and fresh shoot biomass showed a significant reduction along the crop cycle, reaching the same values when lettuce was irrigated with conventional water.

Furthermore, simulated values were estimated based on data obtained experimentally within the predetermined interval for each input variable, namely water depth (25 to 125% of ETc) and days after transplanting (14 to 35 DAT), and modelled with fuzzy logic.

Based on the correlation analysis using the data, the proposed fuzzy model showed higher correlation with the data and higher accuracy compared to the multiple polynomial regression analysis. According to Zhang [13], the fuzzy model obtained the most accurate results in the determination of phosphorus uptake by plants. In the same way, Carozzi et al. [71] determined the performance of cropping systems, using corn as case study, and found lower error using fuzzy modeling in comparison to regression analysis. Applications with fuzzy modeling in agriculture have shown results with greater precision, as in the energy savings in a rotary dryer calculated with a fuzzy multivariable control application, which reduced biomass consumption by 9% [72]. With respect to the sustainability of resources in agriculture, precision agriculture has been used by producers to make decisions and optimize resources, and the use of fuzzy logic has made it possible to optimize the combination of these resources in decision-making [73].

In order to optimize the use of irrigation water, fuzzy controllers have been developed to achieve more sustainable management of agricultural areas [74].

The fuzzy models have a higher degree of association with the collected data from the field than multiple polynomial regressions. We were unable to verify these results based on the statistical tests applied, which have otherwise been proved in this setting. For the value of the mean square error, we noticed that for both types of water over the two cycles, the lowest values occurred.

The correlation coefficient was closer to 1 and therefore closer to the collected data. The value of the model’s accuracy also showed the highest value when the value of “d” was calculated according to Wilmott. *p* value was also determined to verify the significance of the models. In all analyzed cases, *p* value < 0.005.

Carozzi et al. [71] used the fuzzy model for the determination of corn response producing and found that the least error occurred when compared with regression analysis.

According to Zhang et al. [75], the fuzzy model obtained the most accurate results in determining phosphorus absorption by plants.

Polat et al. [76] found that the application of fuzzy logic allowed them to determine more precisely the areas with contamination risks. Weber et al. [77] observed that the fuzzy model applied to the determination of corn hardness yielded results that are more accurate.

## 5. Conclusions

Irrigation using magnetically treated water produced higher development in lettuce over two cycles in comparison to irrigation with conventional water.

A possible reduction in the volume of water consumption to achieve the same production was estimated when lettuce was irrigated with untreated water. In this way, this technology may lead to an increase in food production.

The developed fuzzy model showed a better statistical model adjustment when compared to the multiple polynomial regression based on its association with the data obtained experimentally, with a reduction in the medium square error and an increase in Pearson’s correlation (r) and in the Willmott index (d). Fuzzy modeling provides less adjustment error for curves, presented as a model of behavior analysis of the experimentally tested variables in the field of agricultural sciences. The model developed was adjusted to the conditions of the proposed experiment, so a limitation of the model is that values outside the intervals of the fuzzy sets cannot be used to make inferences about the effects on the lettuce crop. As an agronomic conclusion, the model can help make decisions about irrigation management and the type of water used.

## Figures and Tables

**Table 1:** Combinations of input variables with membership degree of 1 associated with fuzzy sets to build the base rule.

Irrigation Level (IL)		Days after Transplanting (DAT)	
Fuzzy Sets	Point with 1 Degree of Membership Associated	Fuzzy Sets	Point with 1 Degree of Membership Associated
DAT 1	14	IL 1	25%
DAT 1	14	IL 2	50%
DAT 1	14	IL 3	75%
DAT 1	14	IL 4	100%
DAT 1	14	IL 5	125%
DAT 2	21	IL 1	25%
DAT 2	21	IL 2	50%
DAT 2	21	IL 3	75%
DAT 2	21	IL 4	100%
DAT 2	21	IL 5	125%
DAT 3	28	IL 1	25%
DAT 3	28	IL 2	50%
DAT 3	28	IL 3	75%
DAT 3	28	IL 4	100%
DAT 3	28	IL 5	125%
DAT 4	35	IL 1	25%
DAT 4	35	IL 2	50%
DAT 4	35	IL 3	75%
DAT 4	35	IL 4	100%
DAT 4	35	IL 5	125%

**Table 2:** Coefficients of regression and determination of the multiple polynomial equations for growing lettuce parameters: leaf number (LN); fresh shoot biomass (FSB); dry shoot biomass (DSB); fresh root biomass (FRB) and dry root biomass (DRB), sampled at 14, 21, 28 and 35 days after transplanting—DAT, for two cycles (1st and 2nd). Lettuce was submitted to different irrigation levels (25, 50, 75, 100 and 125% of crop evapotranspiration—Etc), and two types of water (magnetically treated water (MW) and conventional water (CW).

Variable	Cycle	$y=\beta_{0}+\sum_{j=1}^{2}\sum_{i=1}^{3}a_{ij}x_{j}^{i}$							R^2^
		$\beta_{0}$	$ax_{1}$	$ax_{1}^{2}$	${ax}_{1}^{3}$	$a_{1}x_{2}$	$a_{2}x_{2}^{2}$	$a_{3}x_{2}^{3}$	
LN-MW	1st	30.33 *	−0.184 *	0.002 *	−0.000006	−0.184 *	0.1 *	0.00068	0.98 *
	2nd	−23.71 *	0.47 *	0.0074 *	−0.000033 *	4.92 *	−0.19 *	0.0027 *	0.89 *
LN-CW	1st	30.4 *	−0.224 *	0.0027 *	−0.00000092	−2.75 *	0.1 *	−0.00059	0.94 *
	2nd	−43.23 *	6.86 *	0.12	−0.31	−0.002 *	0.005 *	0.000001 *	0.94 *
FSB-MW	1st	−133.8 *	−4.58 *	0.082 *	−0.00039 *	35.59 *	−2.17 *	0.0443 *	096 *
	2nd	−122.75 *	0.523	−0.0151 *	0.000098	22.36 *	−1.41 *	0.03 *	0.84 *
FSB-CW	1st	56.59 *	12.7 *	0.2	−0.00089	22 *	−1.171 *	0.024	0.95 *
	2nd	−159.35 *	1.77 *	−0.031 *	0.00016 *	21.89 *	−1.25	0.026	0.91 *
DSB-MW	1st	9.16 *	−0.19	0.0032 *	0.00014	−0.75 *	0.019 *	−0.000015	0.92 *
	2nd	10.08 *	−0.47 *	0.0073 *	−0.000032	−0.35 *	0.0083 *	0.0002 *	0.89 *
DSB-CW	1st	2.11 *	0.019 *	−0.00065	0.00000460891	−0.21 *	−0.001 *	0.00035	0.94 *
	2nd	9.84 *	0.094	−0.0016	0.0000084 *	−1.68 *	0.0718	−0.00074 *	0.88 *
GRB-MW	1st	−23.66 *	−0.13 *	0.0023 *	0.0033 *	4 *	−0.2 *	−0.000011 *	0.94 *
	2nd	−13.69 *	−0.24 *	0.0043 *	−0.00002 *	2.71 *	−0.13 *	0.0023 *	0.92 *
GRB-CW	1st	−9.74 *	0.093	−0.0015	0.0000082 *	1.19 *	−0.061	0.0011 *	0.85 *
	2nd	−6.37 *	0.065 *	−0.0011	0.0011	0.94 *	−0.054 *	0.0000059	0.85 *
DRB-MW	1st	−3.43 *	−0.022	0.00035 *	−0.0000016 *	0.62 *	−0.032 *	0.00054 *	0.86 *
	2nd	2.27 *	−0.029 *	0.00046	−0.0000021	−0.15 *	0.0032 *	0.000013	0.84 *
DRB-CW	1st	−1.43 *	0.0019	−0.00000274 *	0.00000017	0.22 *	−0.012	0.00023 *	0.93 *
	2nd	−0.44 *	0.017 *	−0.00023	0.000001	0.10 *	−0.009	0.0002 *	0.73 *

$x_{1}$: irrigation levels (Etc %); $x_{2}$: samplings (days after transplanting—DAT); * significant for α=5%.

**Table 3:** Association intensity analysis using three criteria: medium square error (MSE); Pearson’s correlation (r) and the Willmott et al. [51] index (d) of fuzzy models and multiple polynomial regression (MPR) for growing lettuce parameters: leaf number (LN); fresh shoot biomass (FSB); dry shoot biomass (DSB); fresh root biomass (FRB) and dry root biomass (DRB), sampled at 14, 21, 28 and 35 days after transplanting—DAT, for two cycles (1st and 2nd). Lettuce was submitted to different irrigation levels (25, 50, 75, 100 and 125% of crop evapotranspiration—ETc), and two types of water (magnetically treated water (MW) and conventional water (CW)).

Growing Lettuce Parameters	Variable	Model	Cycle					
	Water Type		1st			2nd		
			MSE	r	d	MSE	r	d
LN	MW	*fuzzy*	1.20	0.99 *	0.986	1.86	0.96 *	0.999
		*MPR*	1.46	0.98 *	0.773	2.15	0.94 *	0.998
	CW	*fuzzy*	4.44	0.98 *	0.947	7.53	0.95 *	0.998
		*MPR*	5.61	0.83 *	0.932	50.67	0.73 *	0.978
FSB	MW	*fuzzy*	385.68	0.98 *	0.967	912.11	0.96 *	0.955
		*MPR*	423.46	0.96 *	0.523	944.75	0.93 *	0.090
	CW	*fuzzy*	248.56	0.98 *	0.999	993	0.95 *	0.998
		*MPR*	426.06	0.96 *	0.212	1053.7	0.93 *	0.753
DSB	MW	*fuzzy*	1.03	0.97 *	0.771	0.97	0.98 *	0.975
		*MPR*	1.09	0.96 *	0.764	1.02	0.96 *	0.9
	CW	*fuzzy*	0.24	0.97 *	0.89	0.49	0.98 *	0.97
		*MPR*	0.25	0.94 *	0.03	0.80	0.96 *	0.13
FRB	MW	*fuzzy*	1.26	0.98 *	0.87	0.74	0.98 *	0.74
		*MPR*	1.77	0.94 *	0.09	1.62	0.96 *	0.23
	CW	*fuzzy*	1.01	0.96 *	0.94	0.56	0.97 *	0.92
		*MPR*	1.06	0.84 *	0.50	0.58	0.95 *	0.08
DRB	MW	*fuzzy*	0.10	0.96 *	0.99	0.01	0.94 *	0.99
		*MPR*	0.14	0.92 *	0.51	0.02	0.92 *	0.41
	CW	*fuzzy*	0.02	0.96 *	0.97	0.04	0.95 *	0.92
		*MPR*	0.03	0.93 *	0.94	0.05	0.90 *	0.89

* significant with α=5%.

## Data Availability

Data are contained within the article.
